# Enhanced solar-driven evaporation and mineral extraction from hypersaline produced water using low-cost microporous photothermal foam

**DOI:** 10.1016/j.heliyon.2024.e29321

**Published:** 2024-04-15

**Authors:** A.G. Agwu Nnanna, Nnenne A. Nnanna

**Affiliations:** a Texas Water and Energy Institute, USA; b College of Engineering, The University of Texas Permian Basin, Midland, TX, 79707, USA; c Midland College Early College High School, Midland, TX, 79705, USA

## Abstract

The beneficial reuse of produced water (PW) holds significant promise to alleviate water scarcity. However, it still suffers major limitations associated with the high cost of treatment due to energy consumption, economics of scale, and the complex physiochemical constituents. PW is a hypersaline (TDS ∼ 250,000 mg/l) oilfield water with bio-species, organic matter, anions, divalent cations, and radioactive elements. A sustainable treatment option is solar-driven floating photothermal evaporation (PTE), a desalination technology implemented for seawater characterized by simpler chemical compositions and low salinity.

In this work, the photothermal evaporator for PW was fabricated using low-cost commercially available charcoal polyurethane foam. The engineered macrochannels and structural alterations created unique pathways for salt extraction and evaporation; and ensured hydrodynamic balance between the rates of capillary flow and evaporation. This novel design mitigated flooding or dry out on the evaporating surface and kept the system running steadily while simultaneously harvesting freshwater and valuable salts.

The key findings from this work are (a) the development of a novel temperature ratio-based method to determine optimum PTE thickness that results in maximum evaporation and thermal localization, (b) the development of the empirical correlation between the rate of thermal localization, evaporation rate, and PTE thickness. It combines the interplay of convection, evaporative flux, conduction, heat capacitance, and thickness on the thermal response of PTE foam to incident solar flux, and (c) experimental evidence revealing efflorescence and subflorescence salt on the evaporating surface and pore, and (d) enhanced evaporation rate of 118 % or 71.6 kg/day-m^2^ of clean water from chemically complex hypersaline produced water. These findings are significant for the engineering design and estimation of the performance of a PTE in a solar-driven evaporation system.

## Highlights

• Engineered macro channels on PTE provide pathways for the simultaneous harvesting of freshwater and valuable salts from produced water.
• We developed the first temperature ratio-based method to determine optimum PTE thickness for enhanced evaporation and thermal localization.
• The rate of thermal localization and evaporation correlates with PTE thickness.
• Enhanced evaporation rate of 118 % from chemically complex hypersaline produced water.

## Graphical abstract

**Figure:**
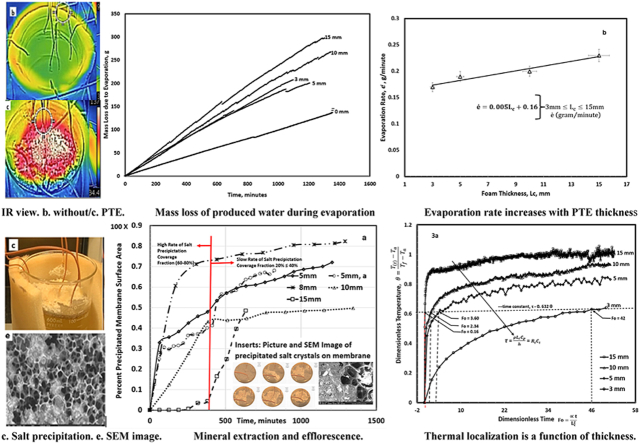

## 1 Introduction

Water scarcity is a major global challenge. Half of the world's population (4 billion people) are experiencing severe water scarcity for at least one month per year [1,2]. A potential new source of water to address the shortage is produced water (PW). It is the most voluminous waste from oil and gas extractions. It was estimated that 3.79 trillion liters of PW were generated in 2017 in the United States [3], and 11 trillion liters globally [4]. Of this quantity, 91 % (10.1 trillion) was disposed to the subsurface into geological formations, 5.5 % surface discharge, and 3.5 % evaporation [5]. Reducing subsurface discharge and expanding the beneficial reuse will alleviate water stress especially in the arid and semi-arid regions. It will mitigate drilling hazards from overpressures in shallow disposal zones [6], potential for induced seismicity [7], and illegal produced water dumping [8]. The key obstacle to treatment of PW for beneficial reuse is energy consumption, economics of scale, and the complex chemical constituents. Produced water contains elevated levels of organic compounds, salts, metals, and radioactive elements that can exhibit salinity several times that of seawater. For example, the total dissolved solids (TDS) concentration is as high as 250,000 mg/l in the Permian Basin [[9], [10], [11]].

Conventional seawater desalination technologies include thermal distillation [12], reverse osmosis [13,14], forward osmosis [15], membrane distillation [16], direct contact membrane distillation [17], and mechanical vapor compression [18]. Depending on water quality requirements, chemical biocides, ultraviolet light, and ozone disinfection are deployed downstream to remove microbial organisms. These technologies, however, are limited to feed water TDS of 30,000–40,000 mg/l [19], roughly six times lower than produced water TDS. Furthermore, they are economically infeasible to treat high TDS waters due to the high osmotic pressure requirements, low permeate flux, high cost of MD modules, and high energy consumption [19,20]. A single effect mechanical vapor compression consumed an estimated 23–42 kWh.m^−3^ for desalination of PW with 260,000 mg/l [19]. Based on the above limitations, it is valuable to explore an energy efficient technology option to lower the cost of treatment.

One such option is renewable energy-based technology. Solar-enabled interfacial evaporation has been applied for seawater desalination [[21], [22], [23], [24], [25], [26]], resulting in simultaneous evaporation and salt extraction [[26], [27], [28], [29], [30], [31]]. It operates by localizing the incident solar on the photothermal interface to enhance evaporation rate [32] and limit heat loss to the bulk water [33]. During evaporation, salt precipitate accumulates on the interfacial surface, blocking the pores, and lowering evaporation. A plethora of studies have been conducted to address the salt accumulation issues [22,[34], [35], [36], [37]]. Nonetheless, most of the investigations have focused on seawater or lake water, characterized by simpler compositions and lower salinity than produced water [38]. The TDS, bio-species and organic matters, anions, and divalent cations in PW, will cause scaling and fouling on the photothermal material and lower the evaporation efficiency. Therefore, further studies are needed to understand these mechanisms and to find specific solutions to control scaling and fouling [34].

An optimization of the photothermal structure thickness is needed to ensure hydrodynamic balance between the rate of capillary flow and salt transport through the pores and the rate of evaporation. This will mitigate flooding or dry out on the evaporating surface. A novel design of localized surface salt precipitation should keep the system running steadily for a long time and simultaneously harvest freshwater and valuable salts [38].

To increase the evaporating surface temperature and thermal localization, techniques such as dispersing nanoparticles in bulk water [39] and interfacial solar evaporation systems [[22], [23], [24], [25], [26],^28^,^29^], have been applied. These strategies reported an increased efficiency up to 90 % in low saline water environment [40]. These techniques have not been implemented in hypersaline produced water in which salts potentially block the water pathway to the evaporating surface and decrease solar-thermal conversion. Furthermore, produced water contains organic and inorganic constituents as well as naturally occurring radioactive materials which constrains solar conversion efficiency. In this paper, photothermal foam samples were placed on the produced water-air interface and exposed to one Sun simulated light source (see inserts in Fig. 2). Four samples of thicknesses: 3, 5, 10, and 15 mm were tested under the same laboratory conditions to determine the optimal thickness relative to evaporation rate. Heat transfer across each sample was governed by an interaction of thermal conduction and convection, capillary flow and solute transport through the pore. During the evaporation process, each foam moved synchronously with the produced water interface relative to the rate of evaporative mass loss. Temperatures at the top and bottom of the foam, bulk water, and the ambient air were detected by a T-type thermocouple and are presented in Fig. 2(a–d).

To the authors’ knowledge, photothermal techniques reported in open literature have not been applied in hypersaline produced water containing organic and inorganic constituents as well as naturally occurring radioactive materials which limits solar conversion efficiency. The focus of this paper is to develop and implement solar interfacial evaporator to treat real-world hypersaline oilfield produced water. The evaporator was fabricated using low cost commercially available charcoal polyurethane foam. It was engineered with macrochannels and surface modifications to reduce efflorescence and subflorescence of salts and enhance evaporation rate. Based on a series of experiments, an optimum foam thickness was determined and correlated with evaporation and salt extraction rates.

## 2 Results

### 2.1 Temperature profiles and thermal localization

**Fig. 1:**
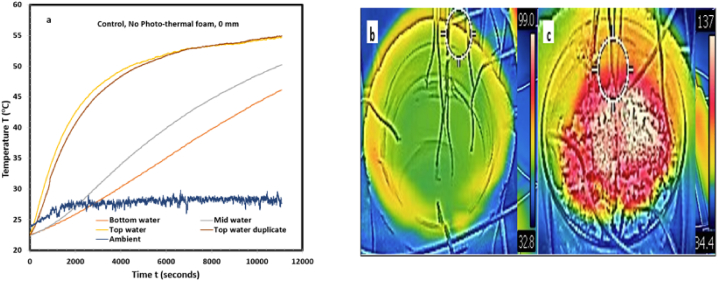
Thermal response of the produced water. (a) Measured temperature of produced water without photothermal foam. It shows continuous increase in the air-water interface temperature and the bulk-water temperature. (b) IR top view of the produced water without the foam. (c) IR top view of the produced water with the foam. Comparison of b and c shows thermal localization (red color) on c. (For interpretation of the references to color in this figure legend, the reader is referred to the Web version of this article.)

Fig. 1a shows that solar evaporation of produced water without photothermal foam gives low energy conversion efficiency. This is due to poor solar absorption at the air-water interface and high transparency of water at visible and near-IR wavelengths. The low absorption coefficient, (0.01 m^-1^) [41,47], leads to slow transient heat response and low thermal localization. Hence, instead of localizing the heat, a large fraction (∼60 %) [42] is transmitted to bulk water for volumetric heating. In Fig. 1a, the bulk temperature increased by 24 °C in 3hrs of operation. Thermal steady-state was not attained indicating progressive conversion of the incident solar flux to sensible heating of the bulk water rather than evaporation. This is further confirmed in Fig. 1b, IR image of the air-water interface without the foam. It reveals low surface temperature when compared with Fig. 1c, with the foam.

**Fig. 2:**
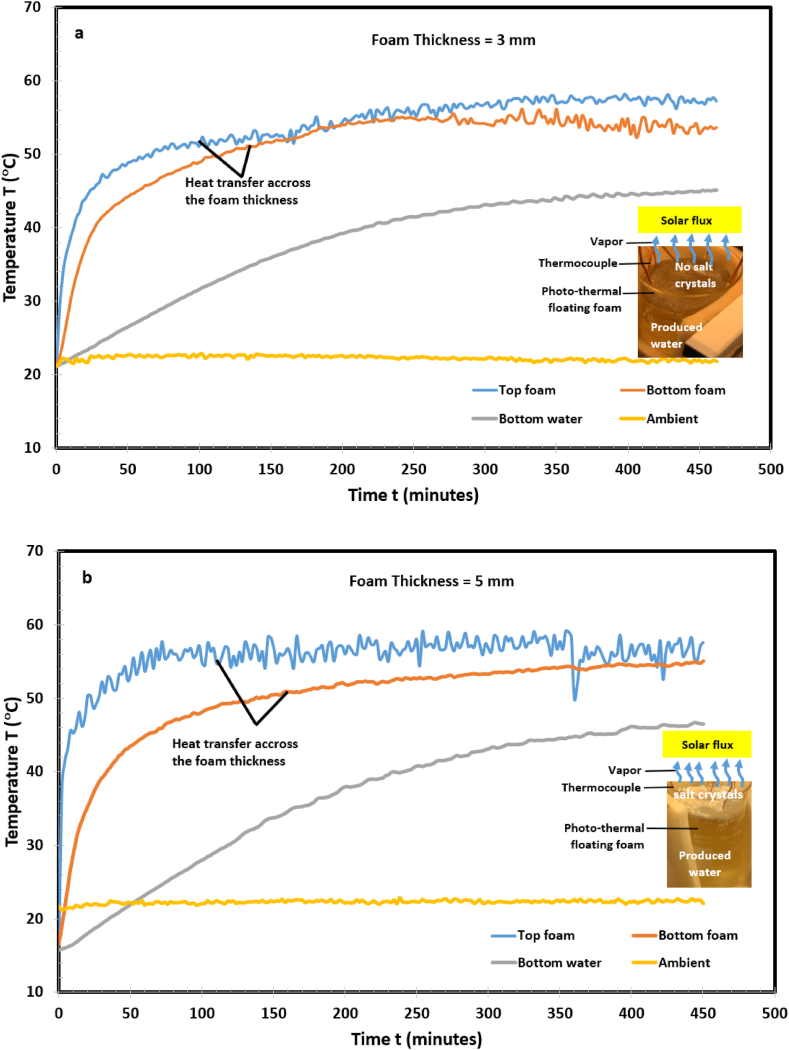

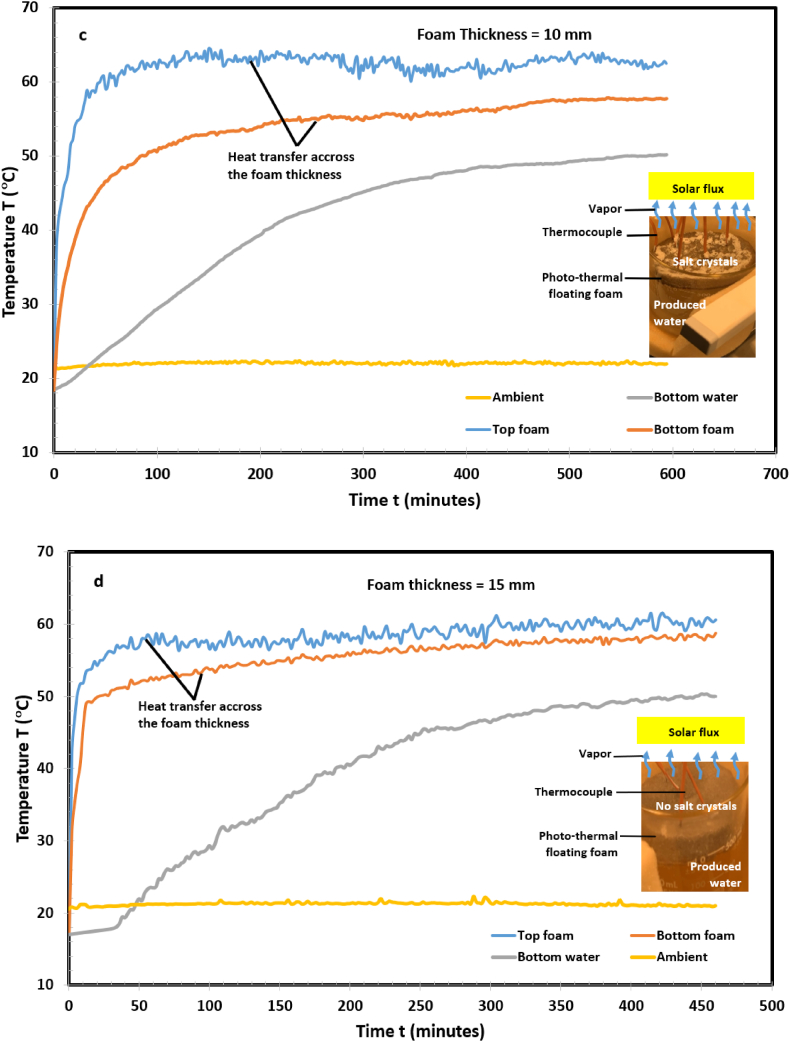
Thermal response of the produced water confined with photothermal foam for (a) 3 mm foam. (b) 5 mm foam. (c) 10 mm foam. (d) 15 mm foam. The 15 mm and 10 mm foams showed rapid response to the incident solar illumination. The top surfaces of the foams reached steady-state temperature conditions in a short time compared to 3 mm and 5 mm.

For brevity, results from Fig. 2(a–d) are summarized in Table 1. It shows that in Fig. 2c (10 mm), the top foam steady state temperature, T_ss, TF_, reached 59 °C in 32 min ($t_{ss,TF})$, while the bulk water, T_ss, BW_, attained 49 °C in 414 min ($t_{ss,BW})$. The subscripts SS, TF, and BW, refers to steady state, top foam, and bulk water, respectively. The ratio of time required to reach thermal equilibrium on the top foam and the bulk water, $t_{ss,TF}/t_{ss,BW}$, was 0.064 at a corresponding temperature ratio of 1.204. The temperature gradient, ΔT, between the top and bottom of the foam was 12 °C. The ΔT signifies thermal resistance to the flow of solar flux to the produced water. A comparison of the data in Table 1 and Fig. 2(a–d) indicates that the 10 mm foam is the optimal thickness. It has more effective thermal localization, high $T_{ss,TF}/T_{ss,BW}$ ratio, low $t_{ss,TF}/t_{ss,BW}$ ratio, and high heat insulation. It offered a balance between water and salt transport by capillary flow through the pores and heat transfer.

**Table 1:** Summary of Fig. 2a–d. A comparison of steady-state temperature and time for photothermal foam thicknesses, 3 mm, 5 mm, 10 mm, and 15 mm.

	Top Foam, TF		Bulk Water, BW		Temperature drops across PTFF		
Thickness (mm)	T_SS_, _TF_ °C	$t_{ss,TF}$, minutes	T_SS_,_BW_ °C	$t_{ss,BW,}$ minutes	ΔT, °C	$T_{ss,TF}/T_{ss,BW}$	$t_{ss,TF}/t_{ss,BW}$
Fig. 1a (0 mm)	–	–	–	–	–	–	
Fig. 2 a (3 mm)	49	60	44	340	7	1.114	0.176
Fig. 2 b (5 mm)	53	36	45	372	5	1.178	0.097
Fig. 2 c (10 mm)	**59**	**32**	**49**	**414**	**12**	**1.204**	**0.064**
Fig. 2 d (15 mm)	58	36	49	406	6	1.184	0.088

The subscripts SS, TF, and BW, refers to steady state, top foam, and bulk water, respectively. T_ss_ is the steady state temperature and t_ss_ is the time required to attain the steady state temperature. $t_{ss,BW}/t_{ss,TF}$ is the ratio of time to reach steady state temperature in the bulk water and the top foam, respectively.

The inserts in Fig. 2(a–d) show concurrent evaporation and salt rejection on the foam. We observed salt crystal patches covering the surface (efflorescence salt) and salt blocking the pores (subflorescence salt). This demonstrates that the photothermal foam developed in this paper can be used for enhanced evaporation and mineral extraction. To the authors’ knowledge this is one of the few reported experimental works on low-cost commercially available photo-thermal floating foam for mineral extraction from hypersaline oilfield produced water. Many related works in literature used synthetic saline water and seawater [10,22,42].

The thermal response in Fig. 2(a–d) is due to competing effects of thermal convection, evaporative flux, and volumetric heat capacitance. To understand the underlying physics governing these phenomena, assuming negligible heat loss to the surroundings, an energy balance at the air-evaporating surface interface yields

(1) $$\theta =T_{\left(t\right)}-T_{a}/T_{f}-T_{a}=\exp\left(-\left(hA_{s}/\rho A_{s}L_{c}C_{p}\right)t\right)$$

where θ is the dimensionless top foam temperature, $T_{\left(t\right)}\text{,}$ $T_{a}\text{,}$ and $T_{f}$ are the respective evaporating surface ambient, and final surface temperatures; *h* is the heat transfer coefficient, $A_{s}$ is the evaporating surface area, ρ is foam density, $L_{c}$ is the foam characteristic length, $C_{p}$ is the foam specific heat capacity. The parameters $hA_{s}\left(T_{\left(t\right)}-T_{a}\right)$ is the convection heat transfer, ($d\left(\Delta m\right)/dt=\left(\rho A_{s}L_{c}C_{p}dT/dt\right)\text{,}$ is the evaporative heat flux due to temperature difference between the top foam and the ambient. The parameter, d(Δm)/dt, is evaporative mass loss and was measured experimentally. A similar formulation to Eq. (1) was reported in Ref. [10]. The quantity $\rho L_{c}C_{p}/h$ is the thermal time constant, τ, which refers to the resistance due to convection and thermal capacitance of the foam.

The combined effect of thermal conduction through the foam thickness and its volumetric heat capacitance is expressed as dimensionless time, Fourier number,

(2) $$Fo=\left(kA_{s}dT/L_{c}\right)/\left(\rho A_{s}L_{c}C_{p}dT/dt\right)=\propto t/L_{c}^{2}$$

where kis the effective thermal conductivity of foam, $A_{s}\;\text{is}$ the surface area, $L_{c}$ is the characteristic length, ρ is the produced water density, $C_{p}$ is the specific heat capacity of produced water, dT is the temperature gradient across the foam, and ∝ is the thermal diffusivity.

**Fig. 3:**
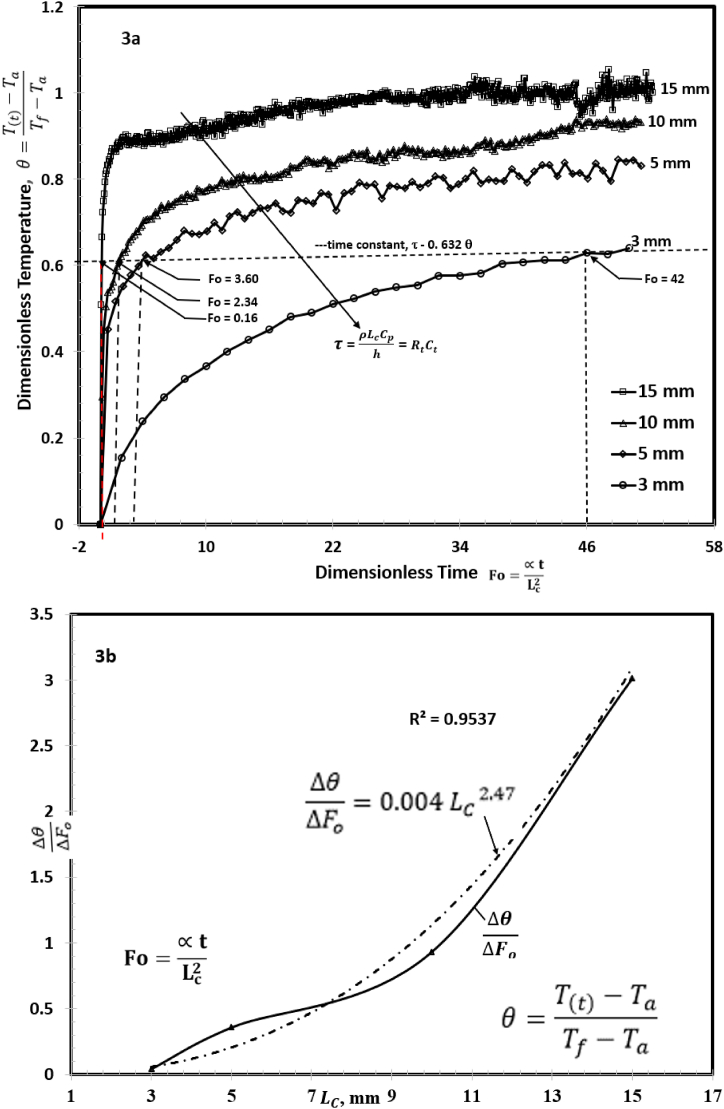
(a) Dimensionless temperature as a function of dimensionless time, Fourier number. It shows the lag time to attain steady state for the various foam thickness. Thermal localization is a function of thickness. (b) Slope of Fig. 3a is plotted as a function of foam thickness.

A plot of θ, (Eq. (1)), as a function of *Fo*, (Eq. (2)), is shown in Fig. 3a. It illustrates the interplay between convection, evaporative flux, conduction, and heat capacitance on the thermal response of the foam to solar illumination. An increase in characteristic length of the foam increased the surface temperature and thermal localization. This is evident for the 10 mm and 15 mm foams. The rapid temperature increase (0≤θ≤0.85) occurred when 0≤Fo≤4, early stages of the heating process in Fig. 3a. Within this range, we performed a linear regression analysis (R^2^ = 84.7 %) for each foam thickness, $L_{C}\text{,}$ to determine the slope, Δθ/ΔFo. The slope is plotted as a function of characteristic length as illustrated in Fig. 3b. It revealed that the rate of thermal localization varies with $L_{C}$ according to the relation, $\Delta \theta /\Delta Fo=0.004L_{C}^{\;2.47}$. The correlation is significant for the design and estimation of the performance of a photo thermal floating foam in a solar-driven evaporation system. It provides a simple method of approximating thermal localization and hence evaporation rate based on the foam thickness.

### 2.2 Mineral extraction

**Fig. 4:**
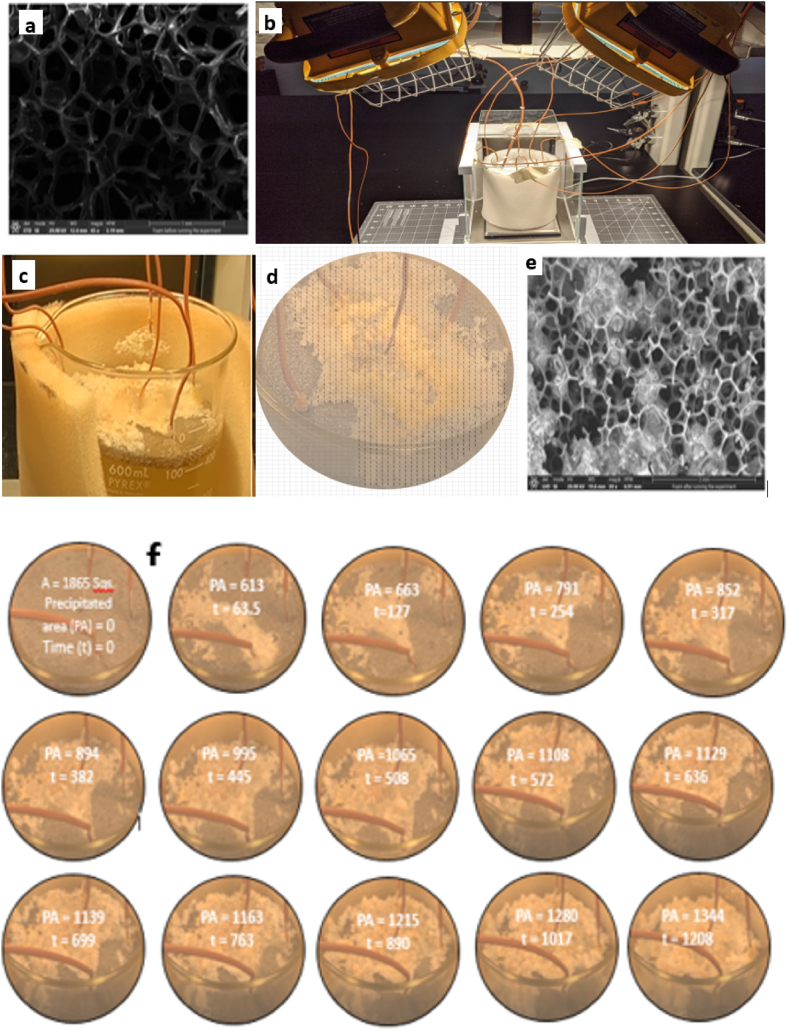
Mineral extraction and temporal evolution of salt coverage area. (a) SEM image of foam before experiment. (b) Experimental setup - simulated solar illumination on the foam. (c) Picture of salt precipitation on the photothermal foam during evaporation (d) Salt precipitates on foam with numerical grid placed over it to determine the total area covered by salt. (e) SEM image of foam after experiment. It shows salt crystals in the pore and on the surface. (f) Dynamics of salt precipitation coverage on the evaporating surface. It shows that the area covered by salt increased with time as evaporation progressed.

Produced water penetrates the foam pores by capillary suction and drags TDS into the pore matrix. Due to evaporative mass loss, ionic concentration of the produced water increased resulting in vapor-brine interface in the pore space. Fig. 4a shows the SEM image of the foam prior to experiments. The elemental analysis shows it contains mostly carbon and oxygen. Fig. 4b shows the produced water reservoir and the solar simulator. At saturation limit, salt crystals began to precipitate in the pores (subflorescence) and migrate to the evaporating surface (efflorescence), see Fig. 4 c and d, and the SEM image, Fig. 4e. The salt spreads on the foam liquid-vapor interface and covers most of the foam surface area by diffusion and advection mechanisms. Salt crystallization is influenced by the thermodynamic state [43], physio-chemical constituents of the produced water, foam characteristic length and porosity [21]. The salt buildup affects the solar-vapor conversion efficiency by increasing produced water osmotic potential, reducing vapor and liquid matrix permeability, and reducing the vapor diffusion coefficient.

**Fig. 5:**
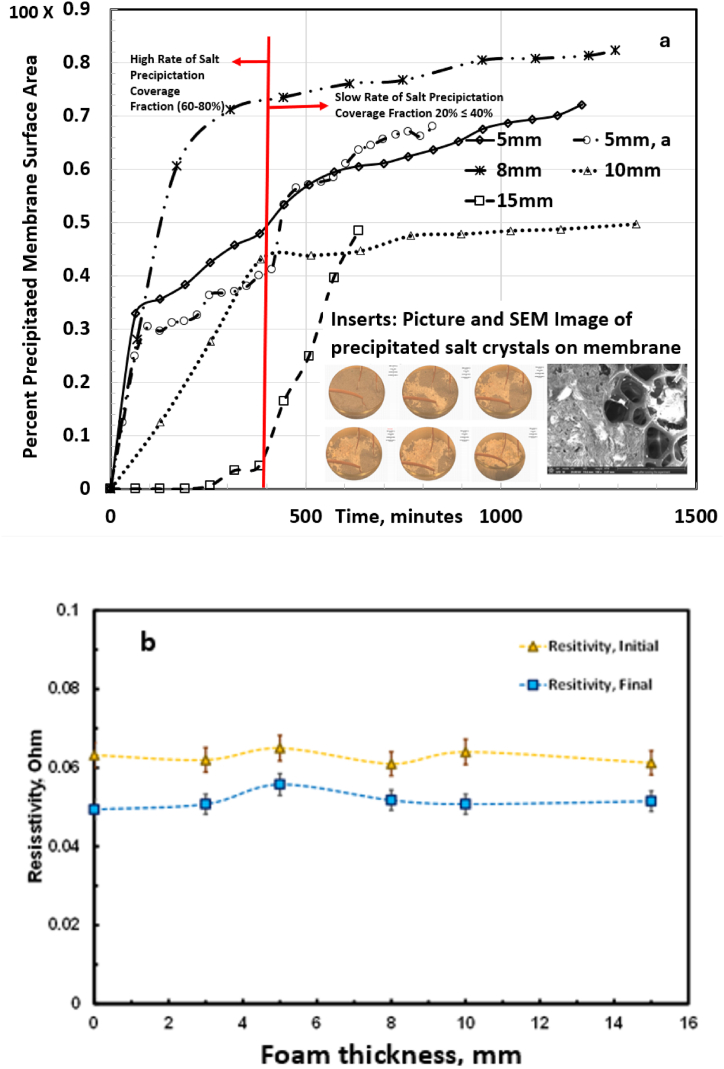
Mineral extraction and temporal evolution of salt coverage area. (a) Variation of salt covered area on the foam surface as a function of time. The high and low precipitation time is demarcated by the red line. The inserts show the picture and SEM image of the salt coverage. (b) Effect of evaporation on salinity of produced water. It shows that salt concentration increases as the water evaporates. (For interpretation of the references to color in this figure legend, the reader is referred to the Web version of this article.)

The dynamics of the salt spreading on the evaporating surface area of each foam, 3, 5, 10, and 15 mm, were video recorded throughout the evaporation operation for further analysis. Fig. 4f shows the progression of the salt (light color) coverage for 5 mm foam. We observed that the rate of salt covered area increased during early stage of evaporation and the salt precipitation rate decelerated over time. To quantify this observation, we systematically placed a numerical grid of 1865 meshes (80 mm^2^) over each image in Fig. 4f. The number of meshes covered by salt precipitates at each time was quantified using an in-house computer algorithm. The light-colored contours in Fig. 4f shows salt precipitated area. The percentage area covered with salt at a given time was calculated and shown in Fig. 5a.

Fig. 5a presents the temporal development of the salt covered area as a function of characteristic length. We make the following empirical observations from Fig. 5a. The inserts show an SEM-EDS analysis with salt crystals in the foam pore. There are two stages of the salt precipitation as demarcated by the red line: (a) stage one signifies high rate of salt coverage as evidenced by the slope. Approximately, 60–80 % of the total salt coverage occurred during this stage. This is attributed to high Peclet number (*Pe* ≫1). Advective mass flow of the ions was dominant resulting in salt accumulation on the foam evaporating surface. (b) Stage two indicates a quasi-steady-state condition in which diffusion was dominant and the rate of the advective mass flow of ion declined. (c) the rate of salt precipitation was inversely related to the evaporative mass loss. We note that the 10 mm and 15 mm had low salt precipitation, high thermal localization, and high evaporation rate. Salt crust on the foam surface blocks the pores where the vapor diffuses. It lowers evaporation due to change in osmotic potential [44]. For the 3 mm, there was no evidence of efflorescence salt precipitation because the foam was slightly submerged and remained hydraulically connected by capillary flow. Hence, it is not represented in Fig. 5a. The plot 5 mm and 5 mm, a, are duplicate measurements.

Fig. 5b compares the initial and final salt concentration in the produced water for each evaporation operation. The final electrical resistivity declined (conductivity increased due to high salt concentration) after each experiment in the range of 14 %–22 %. This indicates that the salt is retained in the produced water rather than transported to the foam pore or the surface. It shows that the structural modifications of the photothermal foam to improve liquid transport, diffusion, and hydraulic connection between the saturated and the evaporation surfaces was effective. The modifications decreased the mass transport of salt into the pore and retained it in the produced water reservoir, thereby enhancing evaporation.

## 3 Enhanced evaporation

**Fig. 6:**
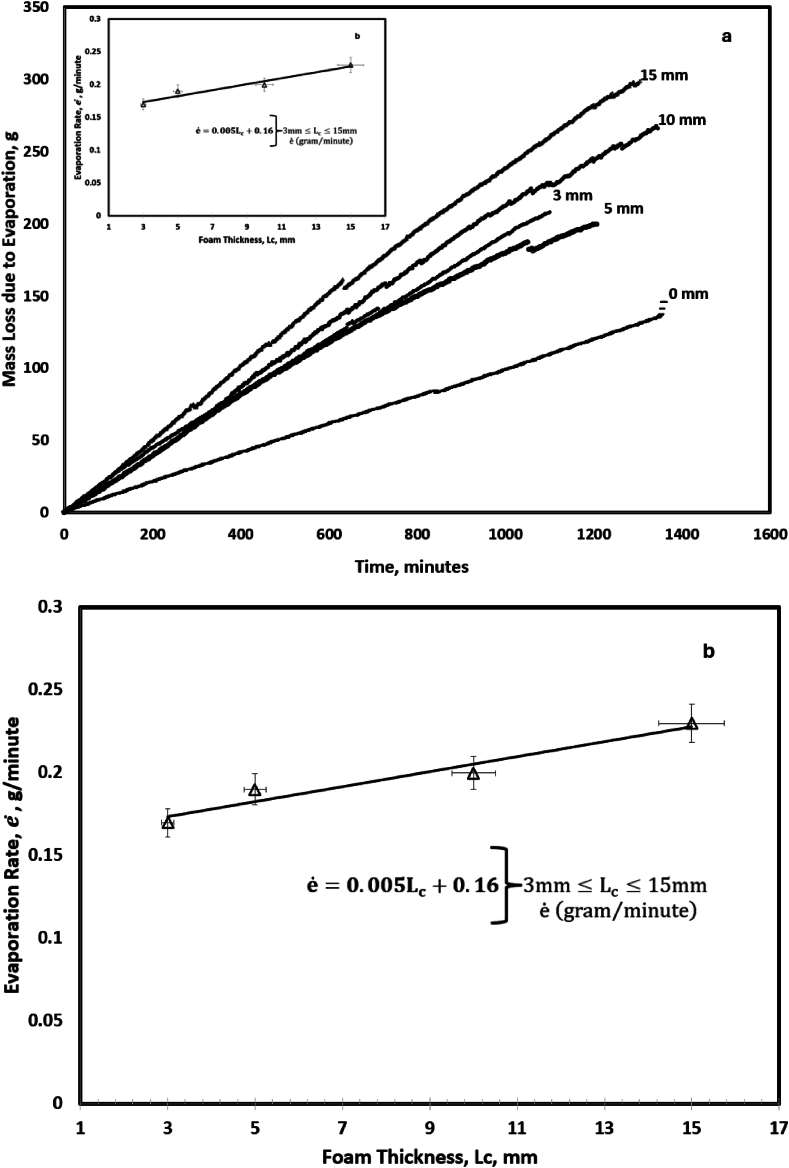
Evaporative mass loss. (a) Mass loss of produced water during evaporation. It compares the performance of the various foam thicknesses. (b) The slope of the mass loss in Fig. 6a for each thickness is plotted in Fig. 6b. It shows a linear increase in evaporation rate with photothermal thickness. A similar trend was noted in Fig. 3b for the thermal localization as a function of thickness.

Fig. 6a shows the mass loss of the produced water reservoir during a 24-h evaporation operation with foam thicknesses (3, 5, 10, and 15 mm) and without foam (0 mm). The reported mass loss in Fig. 6a excludes the contribution due to dark evaporation. Compared with the 0 mm, the 15 mm-thick achieved the highest evaporation rate with an enhancement of 118 %. Higher evaporation efficiencies by 17 % are achievable in larger systems when side walls heat loss of lab-scale reservoir are accounted for [41]. We attribute the high efficiency to insulation of the produced water from volumetric heating and high thermal localization, see Fig. 2d.

The rate of mass loss increased linearly at the rate of 0.23 g/min, 0.20 g/min, 0.17 g/min, 0.19 g/min, and 0.11 g/min for the respective thicknesses, *L*_*c*_, of 15 mm, 10 mm, 5 mm, 3 mm, and 0 mm. The rate of mass loss as a function of foam thickness is shown in Fig. 6b and as an insert in Fig. 6a. A linear fit of the data showed that evaporation rate is related to the foam thickness as $\dot{e}=0.005L_{c}+0.16$. The correlation, $\dot{e}\text{,}$ is needed to analyze Eq. (3)

(3) $$\dot{e}=\frac{\Delta h}{\rho_{pw}L_{pw}}\left\{\frac{\varepsilon \sigma \left(T_{\infty }^{4}\left(r,t\right)-T_{s}^{4}\left(r,t\right)\right)+h_{a}\left(T_{\infty }\left(r,t\right)-T_{s}\left(r,t\right)\right)}{\Delta \delta }-\;K_{eq,}\nabla^{2}T_{s}\left(r,t\right)-{\left(\rho C_{p}\frac{DT\left(r,t\right)}{Dt}\right)}_{pw}\right\}$$

**Fig. 7:**
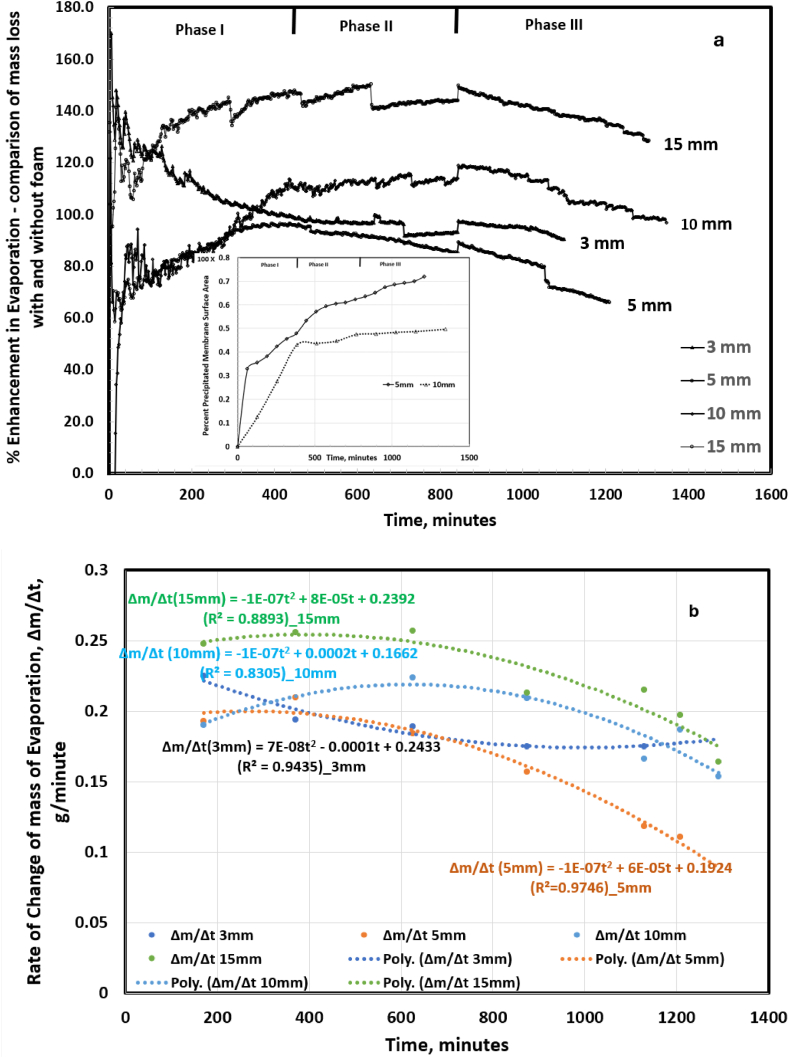
(a) Percentage increase in mass loss due to the foam. For each foam thickness, the percentage enhancement was determined by subtracting mass loss with foam. The plots show three phases of enhancement: Phase I - a rapid linear increase, phase II denotes moderate to quasi steady-state, and phase III indicates a moderate decline. Fig. 7 (b) Rate of change of mass of evaporation with time.

To illustrate the enhancement in evaporation due to confinement of the produced water by the floating foam, the mass loss of produced water without foam was deducted from the mass loss with foam and the results are presented in Fig. 7a. Data in Fig. 7a revealed three phases of evaporation argumentation: phase I was characterized by a rapid linear increase, phase II denotes moderate to quasi steady-state, and phase III indicates a moderate decline. A similar phenomenon was observed in the salt crystallization profile as depicted in the insert in Fig. 5, Fig. 7a. This was attributed to - decline in capillary flow due reduction in capillary pressure between the saturated and evaporating surfaces; pore blockage by salt ions within the foam matrix thus inhibiting vapor diffusion; salt crystallization on the foam surface resulting in an increase in vapor diffusion resistance and decline in evaporation rate; and non-uniform surface temperature distribution due to salt crust growth. Evaporation water loss dominated by vapor diffusion (phase III) shows a stable and low evaporation for saline water evaporation in porous media [45].^.^

Fig. 7b shows the evaporation dynamics as a function of time and characteristic length, Lc, with the 15 mm foam achieving an evaporation rate of 0.23 g/min over a 10-h duration. After which, regeneration of the foam to full evaporation capacity by soaking in water to unclog the pores and dissolve the salt crystals is required. Data in Fig. 7b is useful for practical design of a solar evaporation of produced water using floating foam. In this work, the foam surface area was 0.005 m^2^ and evaporation rate of 360 g/day (0.23 g/min) resulting in a total produced water evaporation of 71570 g/day-m^2^. According to US EPA (2009) the average produced water pond is 4877.41 m^2^ hence the design developed in this paper will produce 349,076 kg/day or 116,359 kg/day-8hr of clean water.

## 4 Conclusion

An experimental investigation of a solar-driven floating photothermal evaporation (PTE), a desalination technology, for treatment of produced water with TDS of 200,000 mg/l, organics, and inorganics constituents is presented.

• PTE with engineered macrochannels and structural alterations created unique pathways for simultaneous salt extraction and evaporation from produced water. It ensured hydrodynamic balance between the rates of capillary flow and evaporation, hence, mitigated flooding or dry out on the evaporating surface and kept the system running steadily while simultaneously harvesting freshwater and valuable salts.
• Photohermal thickness impact evaporative mass loss and mineral extraction. We developed temperature ratio-based method to determine optimum PTE thickness that results in maximum evaporation and thermal localization.
• We developed an empirical correlation between the rate of thermal localization, evaporation rate, and PTE thickness. This correlation combines the interplay of convection, evaporative flux, conduction, heat capacitance, and thickness on the thermal response of PTE foam to incident solar flux.
• Experimental evidence revealed efflorescence and subflorescence salt on the evaporating surface and pore which decreases evaporation.
• We showed an enhanced evaporation rate of 118 % or 71.6 kg/day-m^2^ of clean water from chemically complex hypersaline produced water. These findings are significant for the engineering design and estimation of the performance of a PTE in a solar-driven evaporation system.

## 5 Materials and methods

**Fig. 8a:**
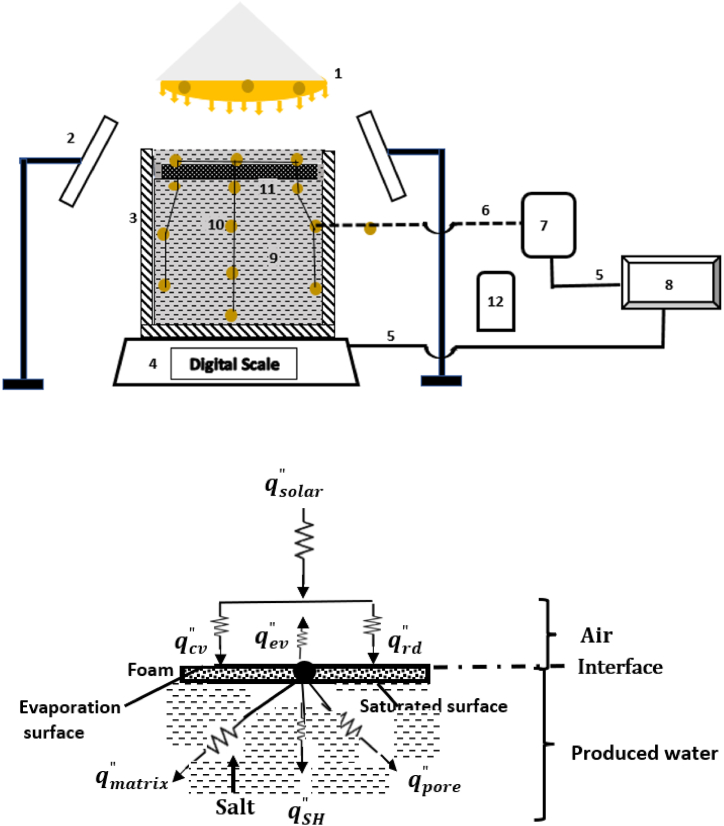
Experimental setup – 1. Solar simulator, 2. iPad mounted on tripod, 3. Insulation, 4. Digital scale, 5. RS 232 cable, 6. Type-J thermocouple wires, 7. Data acquisition system, 8. Personal Computer, 9. Produced water, 10. Thermocouple locations, 11. Photo thermal floating foam, and 12. Humidistat. Fig. 8b. Thermal circuit diagram showing that the incident solar flux arrived on the evaporating surface via convective and radiative heat transfer. The flux contributed to the evaporation and bulk water heating.

**Experimental setup.**Fig. 8a depicts the experimental facility. One sun solar flux, dual head halogen lamp (Luminar WORK, serial 17g 63974), with variable beam angles simulates solar irradiation. The lamp was placed 1 m above the evaporating surface. The produced water reservoir was 88 mm in diameter and 120 mm in depth and was insulated with a 20 mm polyurethane foam. Evaporative mass loss of the produced water was measured by a digital scale (Mettler Toledo-MS1003TS/00). The dark evaporation was measured under the same laboratory conditions without solar illumination. The spatial and temporal variabilities in temperature were measured using Type-J thermocouple and IR camera (FLIR 72001). Two iPads were used to record salt precipitation on the evaporating surface. The temperature, humidity, and mass loss were recorded using data acquisition system (Keysight 34970A). All the measured data and iPad videos were transmitted to a PC for further data reductions. Chemical constituents of the produced water were analyzed using SEM/EDS (Thermo Scientific Prisma, 10104).

**Photothermal foam.** A polyurethane disc (80 mm diameter and thicknesses: 3, 5, 10, and 15 mm) was used as the floating photothermal foam. The commercially available (https://www.uline.com) charcoal foam had incomplete cell walls with pores to enable thermo-fluid transport, and the charcoal color increased thermal absorption. Its material composition enabled water absorption and retention, and moderate swelling when saturated with water. The foam was resistant to chemical and photooxidative degradation under prolonged exposure to ultraviolet radiation. The average porosity was 0.952 ± 0.019, pore diameter was 78.13 μm, thermal conductivity was 0.048–0.05 ± 1.5 % W/mK, and specific heat capacity was 2359–2996 J/kg K [46] (pau et al., 2014). Ninety-six 2.5 mm equally spaced macrochannels were uniformly drilled through the foam using a 3D-printed mold. These channels increased the capillary flow of water and preferential salt transport from the saturated surface to the evaporating surface.

**Produced water.** The produced water was obtained directly from an oilfield midstream water company in the Permian Basin region of the United States. Analytical characterization of the water revealed high level of total dissolved solids, (TDS >200,000 mg/l), which was six times higher than sea water. Furthermore, an elemental analysis of the water samples on mass basis using SEM-EDS showed 38 % carbon, 27 % iron, 9 % sulfur, 3 % silicon, 7 % sodium and chloride, 3 % calcium, and other trace constituents (Al, Mg, Zn, and K). The anions and cations, chloride, sodium, and calcium are predominantly present in produced water with a sublimed strontium, barium, magnesium, bromide, low carbonate, and sulfate^36^. The presence and spatial variability of organics and inorganics, and the high-level of TDS in produced water is a major technological treatment challenge for beneficial reuse and recycle.

**Thermal circuit.**Fig. 8b shows the thermal circuit diagram of the experimental setup. The solar flux illumination arrived at the evaporating surface via convective ($q_{cv}^{\prime \prime })$ and radiative ($q_{rd}^{\prime \prime })$ heat transport. It in turn was used for evaporation ($q_{ev}^{\prime \prime })$, heat conduction ($q_{cd}^{\prime \prime })$ across the foam, and volumetric heating ($q_{SH}^{\prime \prime })$ of the produced water. This is expressed as

(4) $$q_{cv}^{\prime \prime }+q_{rd}^{\prime \prime }=q_{ev}^{\prime \prime }+q_{cd}^{\prime \prime }+q_{SH}^{\prime \prime }$$

where $q_{cd}^{\prime \prime }=q_{matrix}^{\prime \prime }+q_{pore}^{\prime \prime }$; $q_{matrix}^{\prime \prime }$ is heat transfer to the foam material, $q_{SH}^{\prime \prime }$ is the heat transfer to the produced water resulting in temperature change, and $q_{pore}^{\prime \prime }$ is the heat added to the fluid within the foam pore. Assuming negligible $q_{pore}^{\prime \prime }$ and $q_{matrix}^{\prime \prime }$ and rearranging Eq. (4), the energy balance is written as

(5) $$\frac{h_{a}\left(T_{\infty }-T_{s}\right)+\varepsilon \sigma \left(T_{\infty }^{4}-T_{s}^{4}\right)}{\Delta \delta }={\frac{\rho_{pw}\dot{e}L_{pw}}{\Delta h}+k\nabla^{2}T_{s}\left(r,t\right)+\left(\rho C_{p}\frac{DT}{Dt}\right)}_{pw}$$

where σ is the Stefan‐Boltzmann constant (5.67 × 10−8 W/m^2^K^4^), ε is surface emissivity, $T_{\infty }$ is the ambient temperature in Kelvins, ha is the convective heat transfer coefficient to air in W/m^2^K, Cp is the heat capacity in J/kg, k is the thermal conductivity in W/mK, ρ is the density (kg/m^3^), Lpw is the latent heat of vaporization of the produced water (2450 J/kg), $\dot{e}$ denotes the evaporation rate in m/s, and Δδ represents a penetrating depth for radiation, convection, and latent heat fluxes, pw is the produced water. The temporal and spatial evolution of the evaporation rate can be described as a function of surface temperature for constant thermophysical properties of h, Cp, K, Δδ, and ρ as

(6) $$\dot{e}=\frac{\Delta h}{\rho_{pw}L_{pw}}\left\{\frac{\varepsilon \sigma \left(T_{\infty }^{4}\left(r,t\right)-T_{s}^{4}\left(r,t\right)\right)+h_{a}\left(T_{\infty }\left(r,t\right)-T_{s}\left(r,t\right)\right)}{\Delta \delta }-\;K_{eq,}\nabla^{2}T_{s}\left(r,t\right)-{\left(\rho C_{p}\frac{DT\left(r,t\right)}{Dt}\right)}_{pw}\right\}$$

Equation (6) illustrates that minimizing the volumetric heating ${\left(\rho C_{p}\frac{DT\left(r,t\right)}{Dt}\right)}_{pw}$ and conduction losses, $K_{eq,}\nabla^{2}T_{s}\left(r,t\right)\text{,}$ enhance the evaporation rate. Analytical or numerical solution to Eq. (6) requires knowledge of ha, Δδ, $T_{s}\left(r,t\right)$, $T_{pw}\left(r,t\right)$, and the effect of salt transport and precipitation in the pore and on the foam surface. Hence, it will require experimental measurements.

**Produced water flow.** The flow of produced water through the foam is described by the Hagen-Poiseuille equation as $Q=\Delta P{\pi r}^{4}/8\mu L_{c}$, where Q is the flow rate in the throat that connects the foam pores, ΔP=2σ/r is the pressure difference between the saturated and evaporation surface, r is the pore radius, σ is the surface tension, μ is the dynamic viscosity, and $L_{c}$ is the pore length or foam thickness. The capillary pressure gradient driving the produced water flow to the evaporating surface must exceed the gravitational head and viscous losses along the flow path with the pore length. The Hagen-Poiseuille equation has been used by researchers to describe fluid flow through a porous media^,^ [37].

**Salt rejection.** During evaporation from the saturated foam, convection induced by capillary flow transports salt toward the evaporation surface while diffusion tends to spread the salt homogenously on the surface. The resulting interplay between convection and diffusion affects the dynamics of salt distribution on the foam surface. This is commonly described by the Peclet number *Pe* (ratio of convection to diffusion transport). Both mechanisms are related by the Peclet number, *Pe*, the ratio of advection to diffusion, and is expressed as $L_{c}$ × u/α. Where u is the velocity, and α is the diffusivity. The diffusivity of salt in water (αs ∼ 10^−9^ m^2^s^−1^) is two orders of magnitude lower than that of water vapor in the air (αw ∼ 10^−5^ m^2^ s^−1^) resulting in corresponding differences in Peclet numbers and increased mass transport of salt across the foam characteristic length. When *Pe* ≫1, advective mass flow of the ions is dominant, hence salt will accumulate on the foam evaporating surface, [[47], [48], [49], [50]].

**Experimental Procedure.** The foam was saturated in distilled water for approximately 2 h to degasify entrapped air, thus enhancing water absorption and flow through the pores. The foam was dewatered and placed in the 500 ml produced water reservoir. Thermocouples were attached to the foam in r, θ, and z directions. Additional thermocouples were used to measure ambient, bulk water, and the solar simulator surface temperatures. All the thermocouples were connected to three 20-channel multiplexers embedded in a Keysight data acquisition system (DAQ). The DAQ and precision digital mass balance scale were connected to the computer via an RS232 and USB cable, respectively. The iPads monitored and recorded the dynamics of salt formation and progression patterns of the salt precipitation fronts. A total of 23 experiments were conducted. This included triplicates for the control (produced water without foam), 3 mm, 5 mm, 10 mm, and 15 mm thick foams. The average evaporation operation was 15 h. All tests were conducted under standard laboratory conditions.

## Data availability

All relevant data will be made available upon reasonable request from the authors.

## Code availability

The codes used in this work are available upon reasonable request from the authors.

## CRediT authorship contribution statement

**G. Agwu Nnanna:** Writing – original draft, Supervision, Methodology, Investigation, Funding acquisition, Formal analysis, Data curation, Conceptualization, Project administration. **Nnenne A. Nnanna:** Writing – review & editing, Data curation.

## Declaration of competing interest

The authors declare no competing interests.
